# An allelic variant in the intergenic region between *ERAP1* and *ERAP2* correlates with an inverse expression of the two genes

**DOI:** 10.1038/s41598-018-28799-8

**Published:** 2018-07-10

**Authors:** Fabiana Paladini, Maria Teresa Fiorillo, Carolina Vitulano, Valentina Tedeschi, Matteo Piga, Alberto Cauli, Alessandro Mathieu, Rosa Sorrentino

**Affiliations:** 1 Department of Biology and Biotechnology “Charles Darwin”, Sapienza University, Rome, Italy; 2 Rheumatology Unit, University Clinic and AOU of Cagliari, Cagliari, Italy

## Abstract

The Endoplasmatic Reticulum Aminopeptidases *ERAP1* and *ERAP2* are implicated in a variety of immune and non-immune functions. Most studies however have focused on their role in shaping the HLA class I peptidome by trimming peptides to the optimal size. Genome Wide Association Studies highlighted non-synonymous polymorphisms in their coding regions as associated with several immune mediated diseases. The two genes lie contiguous and oppositely oriented on the 5q15 chromosomal region. Very little is known about the transcriptional regulation and the quantitative variations of these enzymes. Here, we correlated the level of transcripts and proteins of the two aminopeptidases in B-lymphoblastoid cell lines from 44 donors harbouring allelic variants in the intergenic region between *ERAP1* and *ERAP2*. We found that the presence of a G instead of an A at SNP rs75862629 in the *ERAP2* gene promoter strongly influences the expression of the two ERAPs with a down-modulation of *ERAP2* coupled with a significant higher expression of *ERAP1*. We therefore show here for the first time a coordinated quantitative regulation of the two *ERAP* genes, which can be relevant for the setting of specific therapeutic approaches.

## Introduction

The ER-resident aminopeptidases *ERAP*1 and *ERAP2* are ubiquitous, zinc-dependent multifunctional enzymes involved in immune activation and inflammation^1–3^, blood pressure regulation^4,5^ and antigenic peptide repertoire shaping^6–10]^. ERAP1 is a type II integral membrane enzyme, whereas ERAP2 is not. The two aminopeptidases share ~49% sequence homology. *ERAP1* and *ERAP2* genes are both regulated by interferons (IFNs) and Tumour Necrosis Factor-α (TNFα)^11^. Whilst differing for the preferred substrates, they have similar proteolytic activities and might even form heterodimers in order to generate optimal peptide repertoires. When overactive, the ERAPs can destroy the putative HLA class I ligands by reducing their length below the threshold of 8–10 amino acids. Loss of both enzymes is a frequent event significantly associated with the lack of HLA class I surface expression^12–17^. While the general rules governing the role of the *ERAP* genes in antigen processing are well established, several studies suggest that the effects on individual HLA class I alleles and/or epitopes may differ^18^.

The *ERAP1* and *ERAP2* genes reside in the same cluster located on the long arm of chromosome 5 (5q15), where they lie contiguous and oppositely oriented. Both genes are polymorphic with strong linkage disequilibrium across the chromosome 5q15 locus and many functional variants appear to primarily affect their enzymatic activity. In particular, it has been shown that a splicing variant in the *ERAP2* gene (rs2248374)^19^ leads to nonsense-mediated decay (NMD) of the mRNA. Since this variant is maintained by a balanced selection to a frequency of approximately 50%, it follows that 25% of the population fails to express the ERAP2 protein^20^.

GWAS have suggested a role for both *ERAP1* and *ERAP2* in the onset of several immuno-mediated diseases (IMD), such as ankylosing spondylitis (AS), Behçet’s disease (BD), psoriasis (Ps), inflammatory bowel disease (IBD), juvenile idiopathic arthritis (JIA), birdshot chorioretinopathy (BSCR), type I diabetes mellitus (T1D) and multiple sclerosis (MS)^21–29^. In particular, the epistatic interactions observed between *ERAP1* and HLA-B27 in AS, HLA-Cw6 in Ps and HLA-B51 in BD, strengthen the central role of antigen presentation in the so-called “MHC-I-opathy”, although the allelic variants of *ERAPs* specifically associated with each disease may be different^30^. In several studies, *ERAAP* deficient mice resulted in significant changes in the MHC-peptide repertoire thus activating CD8+ T lymphocytes and Natural Killer cells^31,32^. These findings suggest a direct interaction of the two ERAP proteins with precursors of HLA class I epitopes, which affect innate and specific immunological surveillance.

Overall, an impaired activity and expression, especially of *ERAP1*, could determine the production of an “atypical” peptide repertoire that predisposes to IMDs^33^ as well as the failure in rejecting tumours and in fighting pathogens^32,34^.

Although most of the studies have been focused on the function of individual SNPs, it must be considered that extensive linkage disequilibrium within and around the *ERAP* genes does exist^35,36^. Indeed, the combination of the *ERAP* polymorphisms can be termed “allotypes” since they are often co-inherited as complex haplotypes, suggesting that this can be the outcome of a selective pressure by pathogens. The SNPs found associated with AS and with other IMDs, as well as with cervical cancer and other pathological conditions^37–39^, probably have a synergistic effect with other variations present in the same haplotype^40,41^. Although there are examples of a direct effect of single SNPs on enzymatic activity and substrate binding^21,42,43^, the data concerning haplotype combinations are conflicting^41,44–49^. In addition, the functionality of different allotypes may be closely dependent on the quality and quantity of the peptide substrates^42^, as well as on the amount of the enzyme itself. Therefore, in some cases, SNPs associated with pathologies may not be causative of the condition themselves, but in linkage disequilibrium with allelic variants impacting on mRNA transcription, maturation and translation^50^. This could explain why the association between the two *ERAP* genes and the different IMDs may rely on distinct or even alternative polymorphisms harboured by different haplotypes in different ethnic groups^51^. Furthermore, many risk variants in *ERAP1* and *ERAP2* (rs30187, rs27524, rs27434, rs1363907, rs2549794) have been reported as expression quantitative trait loci (eQTL) in most tissues^52,53^ and their association could derive from quantitative variations and/or from linkage with other causal SNPs. To date, however, there are no relevant genetic studies in the *ERAP1* and *ERAP2* gene promoters and very little is known about the mechanisms that regulate their expression.

In this study, we have performed a quantitative analysis of *ERAP1* and *ERAP2* in a panel of (EBV)-transformed lymphoblastoid cell lines (B-LCLs) known to express high levels of these aminopeptidases^15,54^. We found an inverse correlation in the expression level of the two aminopeptidases depending on a genetic variant mapping in the intergenic region. It is noteworthy that this SNP is in strong LD with an *ERAP1* variant found associated with several IMDs.

## Results

### Analysis of *ERAP1* and *ERAP2* expression in B-LCLs

**Figure 1:**
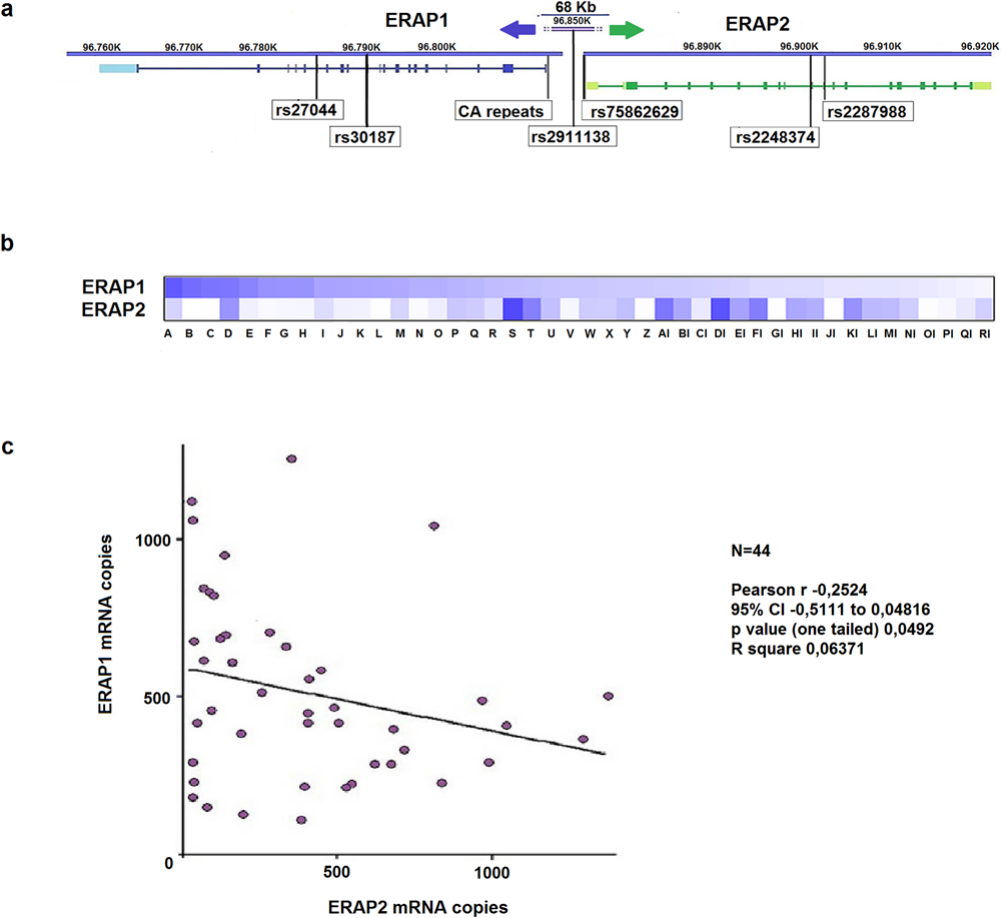
Cartoon illustrating *ERAP1* and *ERAP2* genes organization and location of the polymorphisms analysed in this study and quantification of *ERAP1* and *ERAP2* mRNAs in 44 B-LCLs genotyped for SNP rs2248374. (**a**) SNPs rs30187 and rs27044 are functional variants affecting ERAP1 enzymatic activity; SNP rs2248374 in ERAP2 gene is a “loss of function” variant, in linkage with the “diagnostic” SNP rs2287988 located within the coding region. The other polymorphisms indicated in the cartoon have been analysed in this study. (**b**) Heat map diagrams of *ERAP1* and *ERAP2* expression detected by qRT-PCR analysis in the 44 B-LCLs analysed (A → RI), (**c**) Scatter plot of the data points corresponding to the *ERAP1* and *ERAP2* mRNA quantification. Correlation analysis was performed using Pearson’s r.

A panel of forty-four B-LCLs derived from Sardinian donors, was analysed for *ERAP1* and *ERAP2* gene expression. All B-LCLs were genotyped for SNPs rs30187 and rs27044 in the *ERAP1* gene and rs2248374 in the *ERAP2* gene (Fig. 1a).

The *ERAP1* and *ERAP2* mRNA number of copies was determined by absolute real time PCR (RT-PCR) and then normalized to β-Actin (Fig. S1). As shown in Fig. 1c, regression analysis showed a trend of inverse correlation between the *ERAP1* and *ERAP2* mRNAs (Pearson r = −0.2524; p = 0.049). Interestingly, among individuals heterozygous at rs2248374, 6 out of 24 (Fig. S1) (A → H, except D, hereafter in the text A → H group) showed an *ERAP2* mRNA copies lower than expected whereas *ERAP1* expression was high.

### ERAP2 mRNA and protein expression is regulated by other factors besides rs2248374

**Figure 2:**
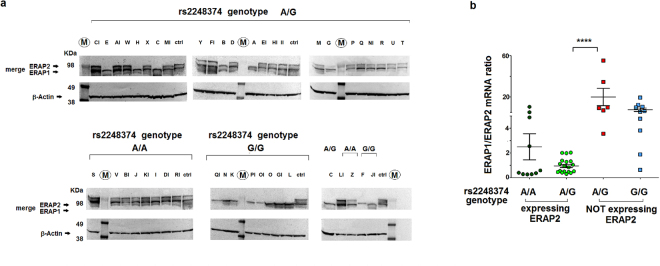
*ERAP1* and *ERAP2* mRNAs and proteins in B-LCLs according to rs2248374 genotype. (**a**) Representative western blots, out of three, displaying ERAP1 and ERAP2 proteins in 44 B-LCLs. The samples were subdivided according to rs2248374 genotype. In the last membrane, besides two A/A and two G/G samples and the ctrl (sample Q), an A/G sample (C) not expressing ERAP2 was also loaded as comparison. M = Molecular Weight protein Marker; ctrl = Q B-LCL. The upper membranes were incubated first with mouse anti-ERAP2 mAb and subsequently with mouse anti-ERAP1 mAb antibody. Full-length gels and blots are included in the Supplementary Information file (Fig. S3). (**b**) Ratio of *ERAP1/ERAP2* mRNAs in B-LCLs genotyped for rs2248374. Horizontal bars represent the mean ± S.E.M. The Mann-Whitney p value from comparison between A/G heterozygous samples expressing and not expressing *ERAP*2 was <0.0001.

Western blot (Fig. 2a) confirmed the mRNA quantitative analysis. Indeed, G/G individuals at rs2248374 did not express *ERAP2* due to the introduction of a premature termination codon (PTC) which induces NMD^20^. However, ERAP2 protein was not detectable in the B-LCLs (A → H group) whereas present in all the other samples genotyped at rs2248374 as A/A or A/G.

Interestingly, the analysis of the *ERAP1* and *ERAP2* transcripts showed that the six B-LCLs heterozygous for rs2248374 and lacking ERAP2 protein, had a higher *ERAP1/ERAP2* mRNA ratio, even above the ratio shown by the G/G homozygous in which the *ERAP2* mRNA encoded by both haplotypes was degraded by NMD (Fig. 2b).

### The presence of the G variant at rs75862629 inversely correlates with *ERAP1* and *ERAP2* transcript levels

To assess whether this variability could be due to *cis*-regulatory variants, the region between the two genes encompassing the TATA-boxes of both promoters was sequenced in three genomic region(Fig. S2). Three polymorphisms were identified: rs75862629 (A/G) in the ERAP2 promoter (region named M2), rs2911138 (C/T) in the M3 region and a “CA” microsatellite in the *ERAP1* gene promoter (100 bp upstream the 5′UTR) (region named M1). As for the CA repeats, we analysed a total of 400 DNA samples from Sardinia and observed a Gaussian distribution of allele frequencies around 32 repeats (data not shown). We therefore arbitrarily named as “L” the alleles with a number of repeats equal or higher than 32 and as “S” the alleles with a number of repeats lower than 32. The frequency in our panel of 44 B-LCLs was consistent with that of Sardinian population (76% “S” and 24% “L”). As for the two SNPs, rs75862629 and rs2911138, their frequency in the Sardinian population was similar to that reported for Italy (https://www.ncbi.nlm.nih.gov/variation/tools/1000genomes/) with 13% G and 77% T, respectively.

**Figure 3:**
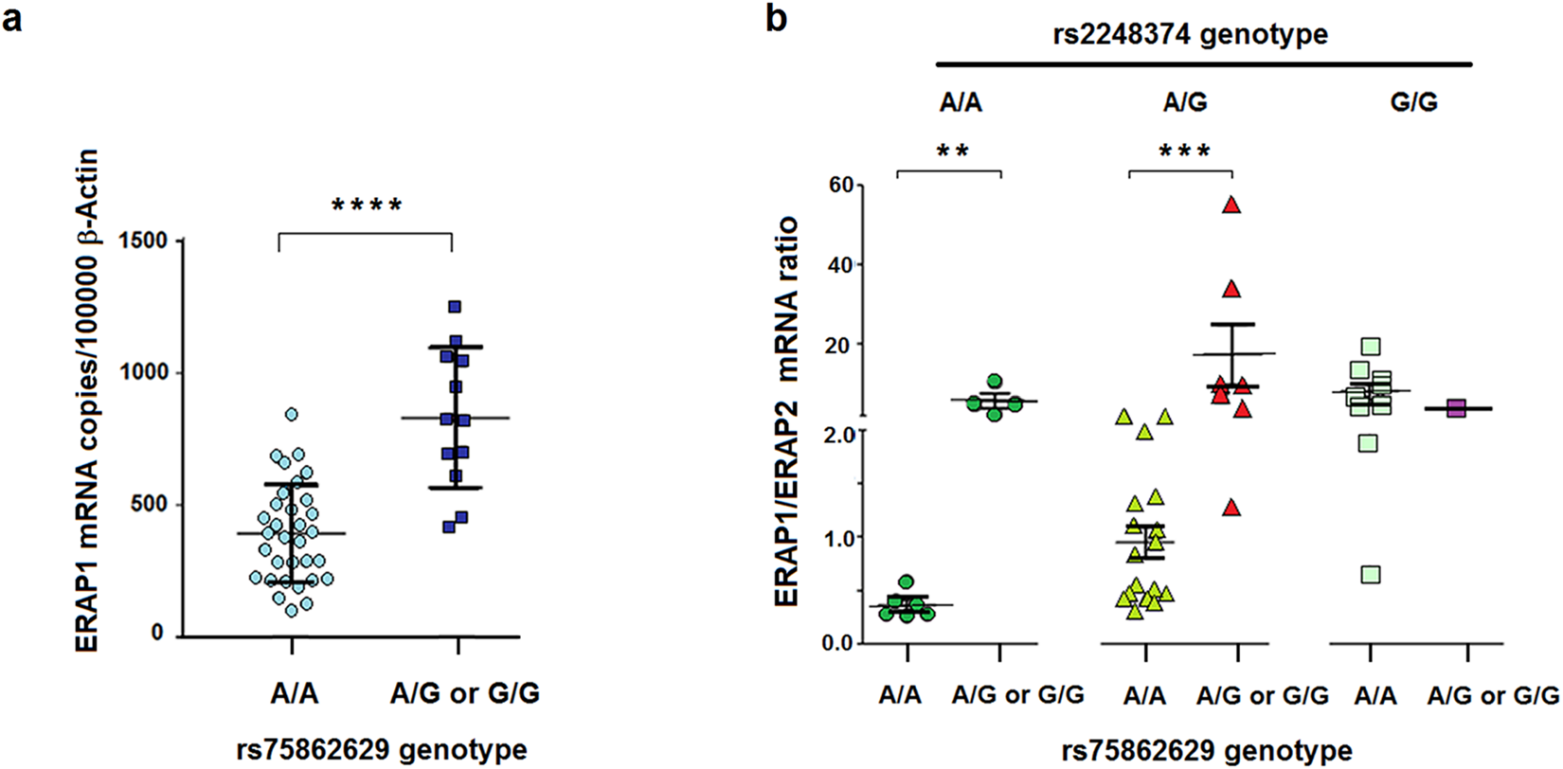
Effect of rs75862629 genotype in the expression of *ERAP1* and *ERAP2*. (**a**) *ERAP1* gene expression correlates with rs75862629 genotype. The absolute expression of *ERAP1* mRNA, as reported in Fig. 2, was analysed in the 44 B-LCLs stratified by the rs75862629 genotype. Horizontal bars represent the means ± S.E.M. ****p value from Mann-Whitney test was <0.0001. (**b**) Correlation of *ERAP1/ERAP2* mRNA ratio with rs75862629 in B-LCLs stratified by the rs2248374 genotype. The samples, subdivided according to the rs2248374 genotype, were analysed for the *ERAP1/ERAP2* ratio depending on the rs75862629 typing. A/G and G/G at rs75862629 were pooled together. All but one of individuals typed as G/G at rs2248374 (not ERAP2 expressors), were A/A at rs75862629. Horizontal bars represent the means ± S.E.M. p value < 0.01 and p < 0.0006 from Mann-Whitney test are indicated by two and three asterisks, respectively.

Allele distribution of rs2911138 and microsatellite showed no correlation with the level of transcripts of either *ERAP1* or *ERAP2*. The G variant at rs75862629 instead, was carried by all those cell lines that, although heterozygous for the functional rs2248374 variant, did not express ERAP2 protein (A → H group, Figs 2a and S3). The other cell lines, I, J and V, which were A/A at the functional ERAP2 rs2248374 and which expressed the expected amount of ERAP2, have been genotyped as A/G at rs75862629, while the Z cell line (A/A at rs2248374) expressing a surprisingly low amount of ERAP2 was indeed G/G at rs75862629. With the exception of the B-LCL, named D, which is heterozygous at both SNPs, all the other ERAP2-expressing cell lines, typed as A/G at rs2248374, were A/A at rs75862629. Only one subject (O) typed as G/G at rs2248374 was also G/G at rs75862629 (Table S2). Therefore, the presence of a G at rs75862629 was consistently correlating with the increased expression of *ERAP1* mRNA (Fig. 3a). The analysis of the *ERAP1/ERAP2* mRNAs ratio was then performed by stratifying the samples according to the functional *ERAP2* rs2248374 genotype. We observed that the G variant at rs75862629 correlated with a higher *ERAP1*/*ERAP2* mRNA ratio regardless of the rs2248374 variant (Fig. 3b).

### ERAP2 transcripts degradation is regulated by additional factors besides SNP rs2248374

**Figure 4:**
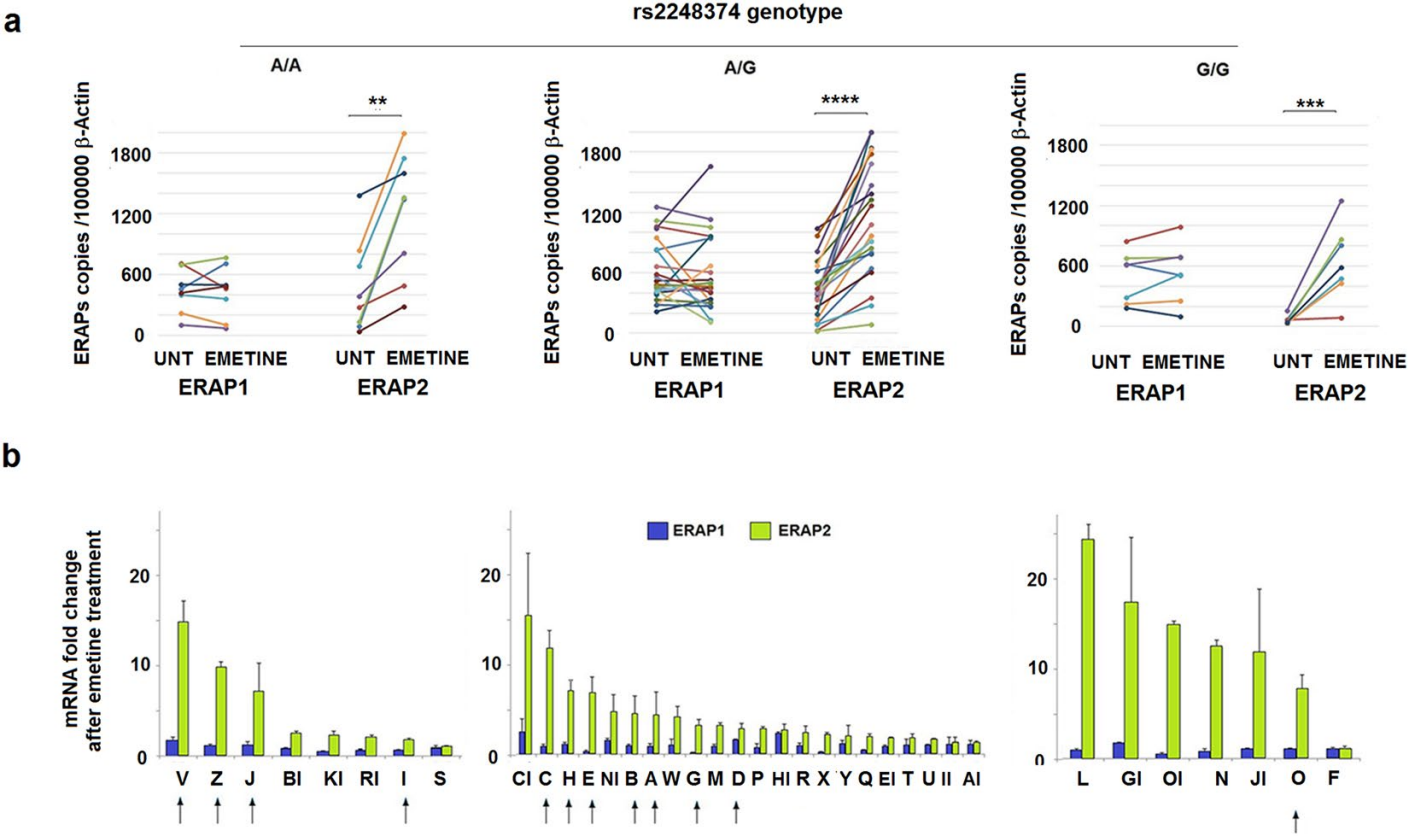
*ERAP2* but not *ERAP1* mRNA is enhanced by NMD inhibition independently from the rs2248374 genotype. (**a**) *ERAP1* and *ERAP2* mRNA copy numbers normalized to 100000 β−Actin copies before and after emetine treatment for 7 h in samples stratified according to rs2248374 genotype. (**b**) ERAPs mRNA fold change (emetine/untreated ratio) of the respective mRNAs. The arrows indicate the presence of G at rs75862629. p value < 0.0054, p < 0.0008, p < 0.0001 obtained by ratio paired t test are indicated by two, three and four asterisks, respectively.

Next we asked whether an increase in the ERAP2 mRNA copy number could directly affect *ERAP1* gene expression. Thirty-seven B-LCLs were treated for 7 hours with emetine, which blocks translation and NMD. We observed that, in the presence of emetine, *ERAP1* mRNA did not undergo any significant variation. Unexpectedly, ERAP2 mRNA expression did increase in all cell lines analysed, even in those B-LCLs typed as A/A at rs2248374 indicating the presence of uncorrected forms of ERAP2 in addition to that marked by G at rs2248374 (Fig. 4a). Following emetine treatment, we observed a higher increase in ERAP2 mRNA in those cells genotyped as A/G or G/G at rs75862629, independently from rs2248374 (Fig. 4b). This observation gives support to the hypothesis that the G variant at rs75862629, mapping in the region between *ERAP1* and *ERAP2* genes, might be in linkage with an additional polymorphism that impacts the correct “maturation” of the transcript inducing, similarly to G at the functional rs2248374, the degradation of ERAP2 mRNA by NMD. The unexpected lack of ERAP2 protein in some samples (A → H group, A/G at rs2248374) and Z (A/A at rs2248374) B-LCLs (see Fig. 2a) can be attributed to G–A/A–G or G–A/G–A rs75862629-rs2248374 haplotype combinations, where the presence of the G at rs75862629 has a similar effect to that of the G at rs2248374. As for “D” B-LCL, an A–A/G–G combination can therefore be predicted.

### The rs75862629 G allele identifies a haplotype correlating with a higher *ERAP1* mRNA and a lower *ERAP2* mRNA expression

**Figure 5:**
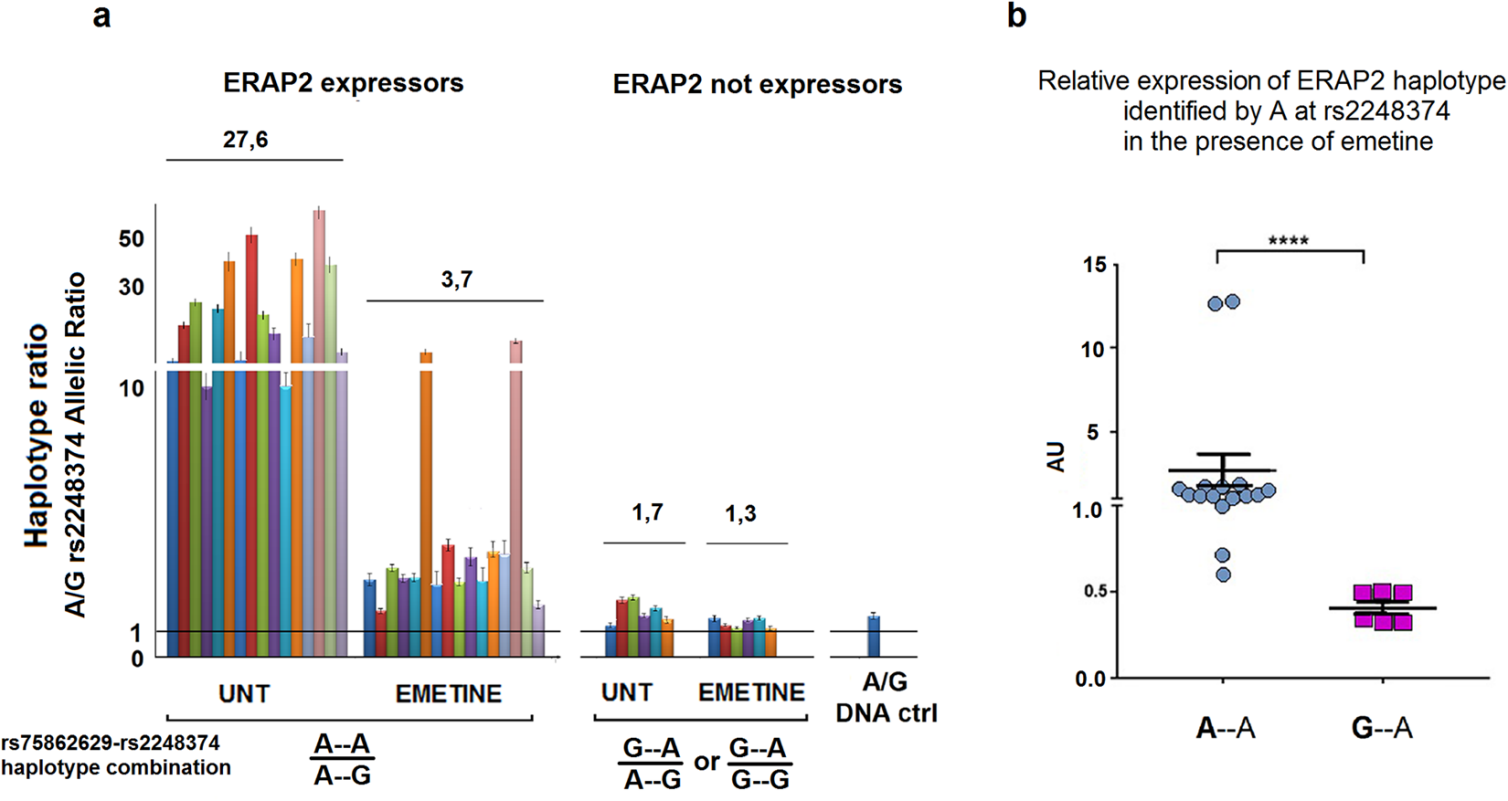
ERAP2 haplotype ratio in rs2248374 heterozygous B-LCLs expressing or not the protein. (**a**) *cDNAs from B-LCLs heterozygous at rs2248374*, *were analysed for the expression of ERAP2 haplotypes in the presence or absence of emetine treatment*. Emetine determined a significant increase in the *ERAP2* A–G haplotype in those cell lines carrying the A–G/A–A haplotype combination at rs75862629 and rs2248374. In contrast, the G–A/A–G haplotype ratio was close to the unit independently from emetine treatment. DNA ctrl represents the haplotype ratio measured in a genomic DNA A/G heterozygous at rs2248374 and at the diagnostic SNP rs2287988 expected to be ~1.0. Histograms represent the mean ± S.E.M. of duplicate of two independent experiments. The average value for allelic ratio among the B-LCLs tested is indicated above each set of bars. (**b**) *Relative expression of A allele at rs2248374 in emetine treated B-LCLs*. cDNA from emetine-treated B-LCLs, heterozygous at rs2248374, were analysed for the expression of ERAP2 haplotypes identified by A variant at rs2248374 (rs75862629-rs2248374 A–A and G–A combinations). The values were normalized to the haplotype identified by the G variant at rs2248374 (rs75862629-rs2248374 A–G). The presence of a G at rs75862629 correlates with a reduced expression of ERAP2. Horizontal bars represent the mean ± S.E.M. The p value from Mann-Whitney test was <0.0001. AU = Arbitrary Units.

To verify whether the haplotype rs75862629-rs2248374 G–A, similarly to the haplotype A–G, determines a lower expression of ERAP2, allele-specific quantitative RT-PCR analysis of heterozygous B-LCLs under NMD inhibition was carried out. Because of the NMD, the SNP rs2248374 is not present in the mature mRNA. Therefore, the expression was monitored using the “diagnostic” SNP rs2287988 (A/G), which falls within the *ERAP2* coding region and presents the variant A in complete linkage with G at rs2248374 and viceversa) (20 and our observation). As expected, a lower level of *ERAP2* mRNA was expressed by the haplotype identified by G at rs2248374 (ratio A–A/A–G > 1). Unexpectedly, the presence of G at rs75862629 makes the haplotypes ratio G–A/A–G or G–A/G–G close to the unit. Furthermore, NMD inhibition induced an increase of the ERAP2 transcript carrying the A–G haplotype of about 8 times, whereas the presence of a G at rs75862629 (G–A or G–G) correlates with a lower susceptible to NMD inhibition (Fig. 5a).

These data indicate that, as to the ERAP2 transcripts, the G allele at rs75862629 tags the haplotypes whose transcripts are susceptible to NMD. However, the greater effect of the NMD inhibition on the haplotype A–G rather than G–A (rs75862629-rs2248374), makes plausible the hypothesis that the G at rs75862629 negatively affects ERAP2 transcription by itself. This was strengthened by a further relative allele specific real-time PCR analysis in which the cDNAs corresponding to G–A and A–A haplotypes from emetine-treated B-LCLs were normalized to the A–G haplotype (Fig. 5b). Cell lines expressing the G–A haplotype (A → H group), produced a lower number of copies compared to the B-LCL expressing the A–A combination. These data were further confirmed when normalized to the β-Actin endogenous control (data not shown).

Interestingly, the comparison of the haplotypes expressed by the two cohorts reported in Fig. 3a, showed that the group of high *ERAP1* expressors carrying the G variant at rs75862629, are preferentially within a haplotype that includes the variant T at rs30187 (p_c_ = 1.5 × 10^−8^) (Table 1) although the LD among the SNPs analysed is not relevant (Fig. S4).

**Table 1:** Frequency of the haplotypes rs75862629-rs2911138-size-rs30187 in the panel of 44 B-LCLs analysed.

Haplotype	Freq.	rs75862629		p_c_
rs75862629-rs2911138-size-rs30187		**A/G + G/G**	**A/A**	
A-T-S-C	0.287	0.086	0.363	0.104
A-T-S-T	0.246	0.170	0.275	3.123
G-T-S-T	0.135	0.494	0.000	1.5 × 10^−8^
A-C-L-T	0.073	0.041	0.085	4.83
A-T-L-C	0.069	0.039	0.079	5.079
A-C-S-C	0.049	0.000	0.067	1.933
A-C-S-T	0.044	0.000	0.061	2.197
A-T-L-T	0.034	0.038	0.033	9.046
G-C-S-C	0.034	0.125	0.000	0.040
A-C-L-C	0.027	0.000	0.037	3.386

rs75862629 A or G; rs2911138 T or C; size: S (Short, <32 CA repeats) or L (Long, ≥32 CA repeats); rs30187 C or T. p_c_ = Bonferroni corrected p value.

## Discussion

In this study, we have analysed the expression of *ERAP1* and *ERAP2* in B-LCLs genotyped for rs30187 (K528R) and rs27044 (Q730E) SNPs in *ERAP1* and rs2248374 in *ERAP2* gene. The analysis of the *ERAP1* and *ERAP2* mRNA levels in the presence or absence of emetine, a NMD inhibitor, pinpointed the SNP rs75862629, mapping in the *ERAP2* promoter, as a marker that correlates with a double effect on *ERAP2* mRNA, that is, lower transcription and higher degradation. Interestingly, this parallels with a higher *ERAP1* transcription suggesting a concerted, opposite regulation.

The presence of T at rs30187 (Lys528) has been found associated with AS, non-AS spondyloarthritis, type I diabetes, psoriasis, multiple sclerosis, anterior uveitis and enthesitis-related arthritis^23^. The major allele C (Arg528) instead reduces *ERAP1* activity and, while protective for AS and psoriasis, is a risk factor for Behçet’s disease. It will be interesting to investigate the interplay between the promoter polymorphism rs75862629 that controls the quantitative level of *ERAP1* and its activity as regulated by rs30187 in different populations and different diseases. Of note in Sardinia, the G allele at rs75862629 is part of a haplotype that includes the T allele at rs30187 and that characterizes individuals with high *ERAP1* expression. This is in agreement with the observation that the T variant at rs30187 correlates with a higher *ERAP1* mRNA transcription and protein expression, particularly in B-LCLs and monocytes-derived dendritic cells^35,36^. Other studies have suggested that *ERAP1* expression traits may be under a *cis*-regulatory control and that rs30187 shows the strongest association with *cis*-regulation tag frequency-derived haplotype frameworks^52,55,56^. Since the lack of *ERAP* expression was not associated with DNA methylation in the promoter of *ERAP1* or *ERAP2*^57^, our findings suggest that this latter phenomenon could be due to the presence of variants that, falling into *cis*-regulatory elements, would favour the hijacking of specific factors towards the *ERAP1* rather than *ERAP2* transcription. Although our study has been limited to few markers and therefore other *cis*-elements can be responsible of the observed phenomenon, it is interesting that the rs75862629 maps within the *ERAP2* gene promoter region flanked by putative TATA-box sequences thus making plausible that rs75862629 itself may affect gene transcription resulting in a modification within the putative transcription factor binding site of IRF-1^1^. In this context, it is remarkable that a constitutively inverse mRNA expression pattern was also found for *ERAP1* and *ERAP2* in melanoma cell lines compared to melanocytes^57^. A further possibility is that *ERAP1* grabs transcription factors that control the expression of both genes and this results in a poor *ERAP2* transcription. A lower production of negative regulators (i.e. non-coding RNA), whose genes could overlap with the *ERAP2* gene is also conceivable. In this regard, the *ERAP2* mRNA, regardless of rs2248374 allelic variant and in contrast to *ERAP1* mRNA, is particularly influenced by emetine treatment, suggesting the existence of a higher number of alternatively spliced mRNA isoforms undergoing NMD degradation.

These findings provide a new tool to evaluate the functional consequences that a higher expression of *ERAP1*, counterbalanced by a lack of expression of *ERAP2*, can have in the shaping of the peptidome and on the numerous functions of these enzymes. It must be considered that the *ERAP*s may influence pathological processes other than those strictly involving cell-mediated immunity. Indeed, *ERAP1* role in angiogenesis and in the regulation of blood pressure arises the possibility that in some IMDs, vascular alterations taking place during inflammation as well as vessel remodelling and stability, can contribute to the pathologies and, in this context, the quantitative variations of the *ERAP1* expression can play a relevant role^58^.

In conclusion, we demonstrated the existence of a regulatory intergenic region marked by the SNP rs75862629 that balances the level of the two *ERAP* genes. Overall, a deeper knowledge of the possible consequences of *ERAPs* quantitative variations, particularly of *ERAP1*, and of factors controlling their expression, may well be fundamental to the design of novel and specific therapeutic approaches in different pathologies.

## Methods

### Ethics

Peripheral Blood Mononuclear Cells (PBMC) were collected from forty-four Sardinian donors recruited in the Department of Medical Sciences, University of Cagliari, Italy. Informed consent was obtained by all subjects and the study was approved by the local ethics committee (365/09/CE, University Hospital, Cagliari, Italy). All experiments were performed in accordance with relevant guidelines and regulations.

### B-Lymphoblastoid cell line generation and culture

PBMC were isolated from 30 ml of freshly drawn blood by Ficoll density-gradient centrifugation (StemCell Technologies). B-lymphoblastoid cell lines (B-LCLs) were established from Epstein-Barr virus (EBV) transformed B lymphocytes according to the standard protocol^59^, and grown in an atmosphere with 5% CO_2_ at 37 °C in RPMI 1640 medium supplemented with 10% heat-inactivated fetal bovine serum, penicillin (100 units/ml), streptomycin (100 μg/ml) and amphotericin B (Invitrogen). B-LCLs were cultured at 5 × 10^5^/ml. For the inhibition of Nonsense Mediated Decay (NMD), cells were transferred to a new flask and kept in culture for 7 h in the presence or absence of emetine (Sigma) at concentration of 100 μg/ml.

### Genotyping

Genomic DNA was obtained from B-LCLs or from EDTA-treated peripheral blood samples using QIAamp DNA Blood mini kit (Qiagen, Hilden, Germany) according to the manufacturer’s protocol. SNPs genotyping was performed by quantitative Real-Time Polymerase Chain reaction (qRT-PCR) with functionally tested TaqMan Allelic Discrimination Assay (rs30187: C_3056885_10; rs27044: C_3956870_10; rs2248374: C_25649529_10; rs2287988: C_25649516_10; 7300 real-time PCR system, Applied Biosystems). The analysis of the genomic regions reported as M1, M2 and M3 (Fig. S2) and the typing of SNPs rs75862629 and rs2911138 were determined by direct sequencing. Briefly, 3 amplicons (1 for each region) were amplified from 100 ng of genomic DNA in a final volume of 30 μl containing: 0.5 μM of each primer (Table S1); 1x PCR Buffer; 200 μM of dNTP mix and 0.8 U of “High Fidelity DNA Polymerase” (New England BioLabs). Cycling conditions were as following: 10 s at 98 °C, 20 s at 57 °C, and 30 s at 72 °C for 35 cycles; followed by a final step of 10 min at 72 °C. PCR products were checked by agarose gel electrophoresis using GelGreen nucleic acid staining (10,000x in water, Biotium). Approximately 40 ng of the purified product underwent sequencing on an ABI 3730xl capillary sequencer (Applied Biosystems) and analysis by Bioedit software.

The number of CA repeats (hereinafter named as “size”) in the promoter region of *ERAP1* gene was determined by fragment length analysis. Briefly, the microsatellite region was amplified by PCR with fluorescent (6-FAM) −5′-end labelled primers (Table S1). PCR products were size-separated and analysed on Applied Biosystems 3730 automated sequencer equipped with GENESCAN software (Applied Biosystems). A standard size of repeats was obtained by cloning the fragment from U937 cells (22 CA).

### Gene expression analysis

Total RNA from B-LCLs was extracted using Trizol Reagent (Thermo Fisher Scientific) according to the manufacturer’s protocol. RNA was quantified by a Nanodrop spectrophotometer ND-1000 (Thermo Scientific) and its quality was assessed by 1% agarose/Tris–Acetate–EDTA (TAE) gel electrophoresis. 1 μg of RNA underwent reverse transcription reaction using High-Capacity cDNA Reverse Transcription Kit (Applied Biosystems).

*ERAP1*, *ERAP2* and β*-Actin* mRNA absolute quantification was performed by qRT-PCR using the standard curve method. RT-qPCR was performed with gene specific primer sets (Table S2) in a singleplex, using the SYBR PrimeScript RT-PCR kit and an Applied Biosystems 7500 Real-Time PCR system (Applied Biosystems; Thermo Fisher Scientific, Inc.). qPCR was performed in 20 µl containing 10 µl of SYBR Green PCR Master mix (Roche Diagnostics), 1 µl cDNA, 100 µM of forward and reverse primers for 45 cycles at 95 °C for 30 sec and 60 °C for 1 min after an initial step at 95 °C for 10′. A dissociation step was included in all reactions to confirm single specific PCR product amplification and to define the Tm for each amplicon. Genomic DNA, reverse-transcription and positive PCRs were analysed to control for genomic DNA contamination, reverse transcription efficiency and polymerase chain reaction, respectively. For absolute quantification, absolute transcript copy numbers for each gene and replicate were calculated with the ABI 7500 system SDS software version 1.4 (Applied Biosystems). Absolute quantification is expressed as mean number of copies (nc) from six amplification values (duplicate of at least three independent experiments) with standard deviation. For absolute quantification with normalization, absolute transcript copy numbers were related to 100000 copy numbers of β-Actin endogenous control.

### Analysis of allele-specific gene expression

B-LCLs heterozygous at SNP rs2248374 were treated with 100 μg/ml of emetine (Sigma) for 7 hours to inhibit NMD^60^. Total RNA was extracted, treated with DNase according to the manufacturer’s instructions (RQ1 RNase-Free DNase, Promega) and used to generate cDNA as already described. Haplotype-specific *ERAP2* cDNA was quantified in triplicate using an allele-discriminating TaqMan genotyping assay for the rs2287988 coding diagnostic SNP^20^. Briefly, we generated a standard curve consisting of serial dilutions of DNA samples homozygous for A or for G allele. We used a heterozygous genomic DNA sample to validate the regression equation, in which a mean allelic ratio of 1.0 was expected.

Relative quantification of rs75862629-rs2248374 G–A and A–A haplotypes after emetine treatment was performed using the TaqMan genotyping assay for the rs2287988 coding diagnostic SNP. The expression level of mRNAs is indicated as ratio of the targets normalized to the A-G haplotype using the 2−ΔΔCt method, as ratios of fold change relative to the calibrator. The 2−ΔΔCt method assumes that the amplification efficiency of the reaction is ideal and constant for each sample.

### Western blot analysis

Approximately 3 × 10^6^ cells were harvested, washed twice in 1x PBS and lysed for 30 min on ice in 30 μl of 1% NP40 protein lysis buffer containing 100 U/ml of phenylmethylsulfonyl fluoride (PMSF), 1 μg/ml of aprotinin and 0.5% sodium deoxycholate proteinase inhibitors. Proteins were obtained from the cell lysates by centrifugation at 16363 *g* for 15 min at 4 °C, and their concentrations determined using the Biorad protein assay kit (Biorad, Hercules, CA) with bovine serum albumin (BSA) used as standard. 40 μg of protein extract for each sample were separated on a 4–12% NuPage Bis-Tris gel (Invitrogen) at 125 V for 100 min in NuPage MES SDS Running Buffer (Invitrogen), and transferred to nitrocellulose membranes. The membranes were incubated ON with mouse anti-ERAP1 mAb antibody (clone B-10, sc-271823 SantaCruz), mouse anti-ERAP2 mAb (clone 3F5, MAB 3830 R&D Systems) and mouse anti-β-Actin mAb (clone C4, sc-477778 SantaCruz). The membranes were washed twice in TBST, incubated with horseradish peroxidase–conjugated secondary Ab (Jackson Immunoresearch Laboratories, Inc. West Grove, PA) and revealed by ECL Western blotting detection system (Amersham). The proteins were visualized by ChemiDoc XRS+ System (Biorad, California, USA).

### Statistics

In the scatter plots the mean ± standard error of the mean (SEM) are indicated. Pearson’s test (one tailed) was employed for the correlation analysis of *ERAP1* and *ERAP2* transcripts. For comparison of two groups, two tailed Mann–Whitney U-test or paired t test were used. Statistical analyses were performed using GraphPad Prism 5 software (GraphPad, San Diego, CA, USA). Haploview version 3.2 (http://www.broad.mit.edu/mpg/haploview/) was used to analyse the patterns of haplotype blocks (*chi square* p values). P values underwent Bonferroni correction for multiple comparisons.

## Electronic supplementary material

Supplementary Information
