# Supplementary material for: An allelic variant in the intergenic region between ERAP1 and ERAP2 correlates with an inverse expression of the two genes
Source: Sci Rep. 2018 Jul 10;8:10398. doi: 10.1038/s41598-018-28799-8 (PMC6039459; doi:10.1038/s41598-018-28799-8)
Supplement: Supplementary file 1 — Supplementary Information [file 41598_2018_28799_MOESM1_ESM.docx]

**An allelic variant in the intergenic region between *ERAP1* and *ERAP2* correlates with an inverse expression of the two genes**

**Fabiana Paladini^1^*, Maria Teresa Fiorillo^1^, Carolina Vitulano^1^, Valentina Tedeschi^1^, Matteo Piga^2^, Alberto Cauli^2^, Alessandro Mathieu^2^, Rosa Sorrentino^1^***

^1^Department of Biology and Biotechnology “Charles Darwin”, Sapienza University, Rome Italy. ^2^Rheumatology Unit, University Clinic and AOU of Cagliari, Cagliari Italy.

*co-corresponding authors: FP and RS (e-mail: [fabiana.paladini@uniroma1.it](mailto:fabiana.paladini@uniroma1.it);<mailto:> [rosa.sorrentino@uniroma1.it](mailto:rosa.sorrentino@uniroma1.it))

**
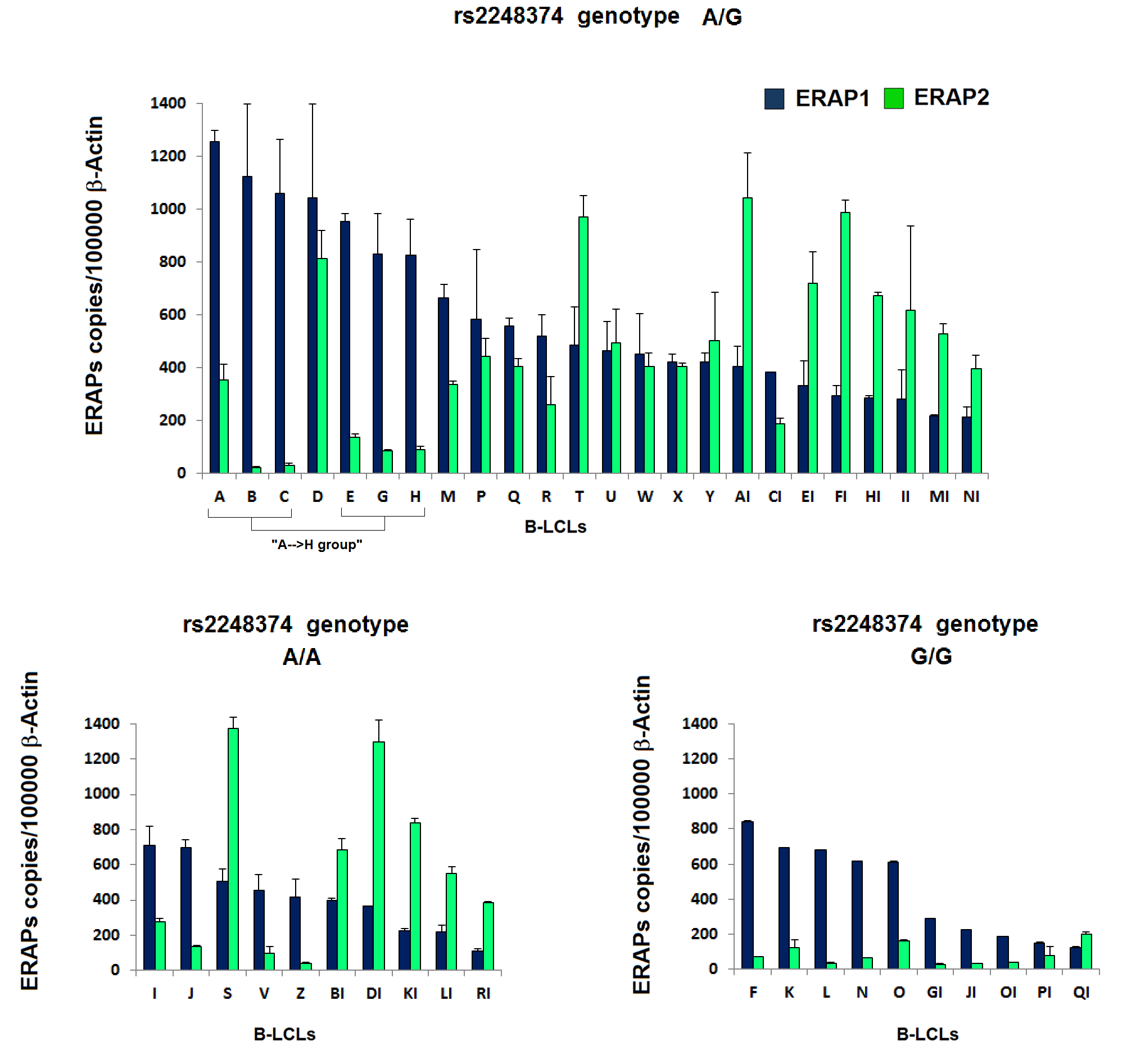
**

**Supplemetary Figure S1.** Barplots represent the mean number of *ERAP1* and *ERAP2* mRNA copies from six amplification values (duplicate of three independent experiments) with standard deviation. Samples are clustered depending on rs2248374 genotype. Absolute transcript copy numbers were normalized to 100000 copy numbers of β-Actin as endogenous control.


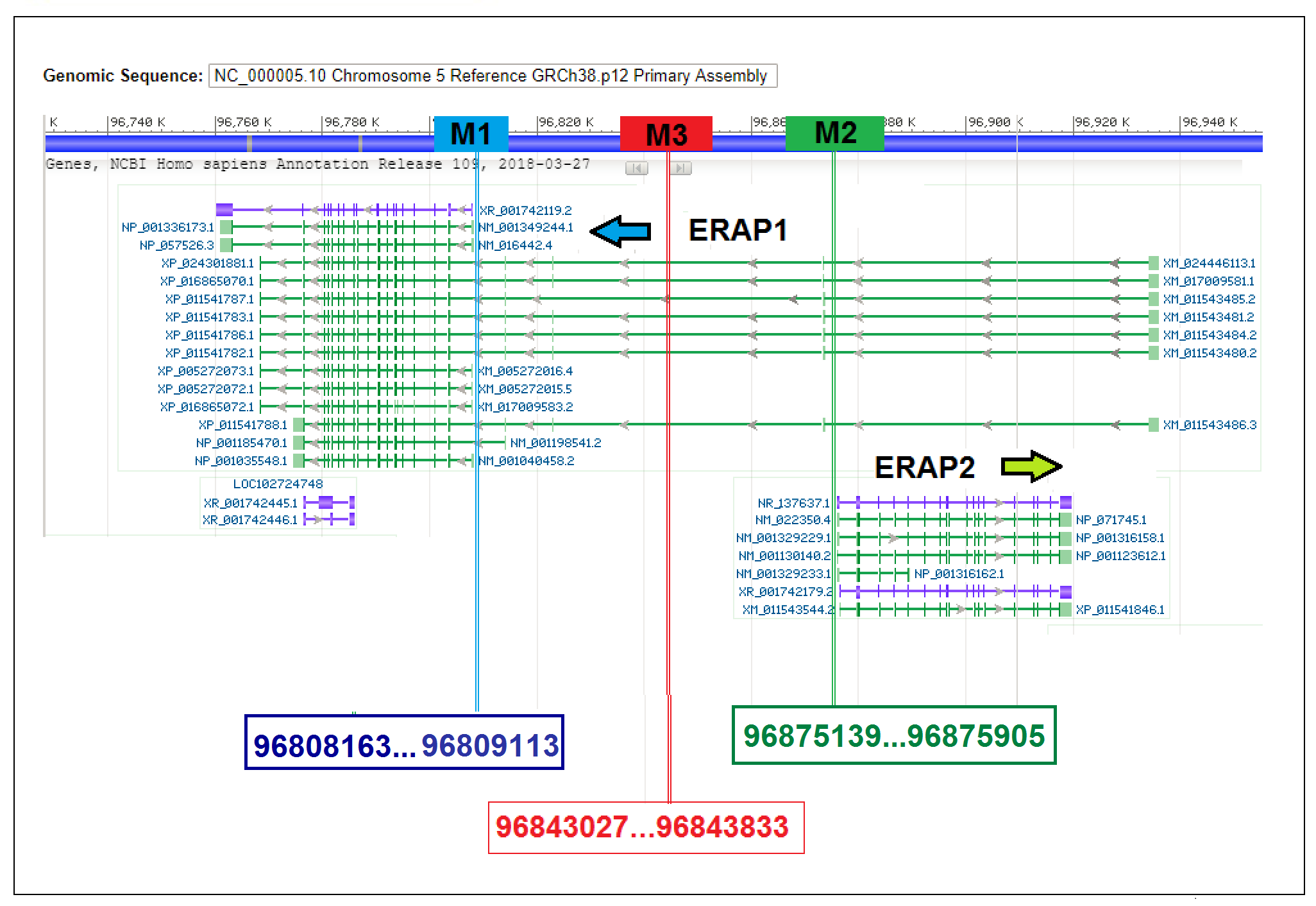


**Supplementary** **Figure S2.** Mapping of the genomic regions sequenced in all 44 B-LCLs under study. NCBI database (http://www.ncbi.nlm.nih.gov/): Homo sapiens chromosome 5, GRCh38.p7, Primary Assembly; 96962493-96732733 (230 Kbp) bases shown. M1, M2 and M3 indicate the three regions sequenced in all 44 DNA samples.


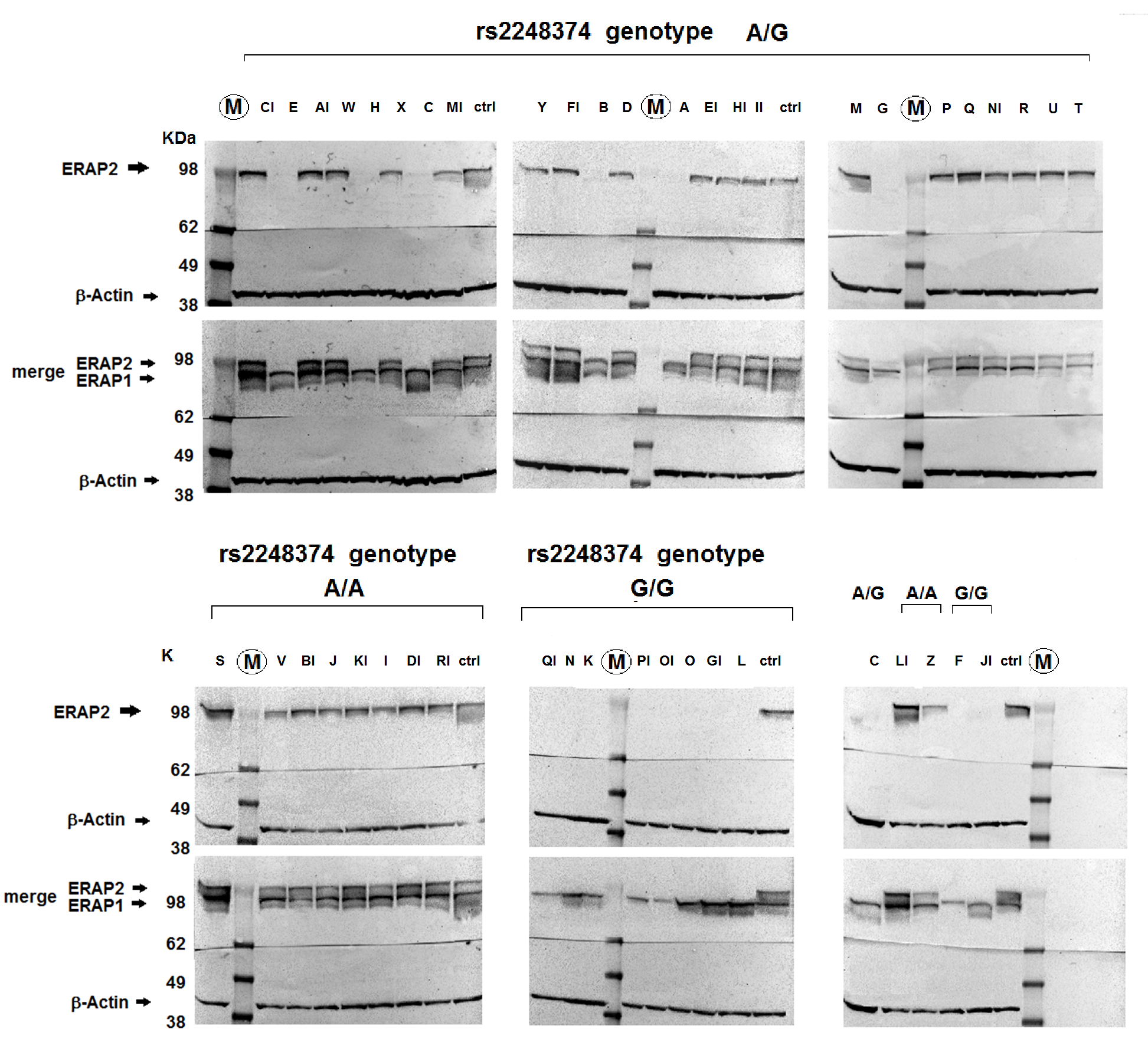


**Supplementary Figure S3.** Full-length gels and blots shown in Fig.2a. SDS-PAGE was 4–12% gradient (NuPage Bis-Tris gel; Invitrogen). Nitrocellulose membranes were cutted at 62 KDa molecular weight. The upper membranes were incubated ON first with mouse anti-ERAP2 mAb (clone 3F5, MAB 3830 R&D Systems) and subsequently with mouse anti-ERAP1 mAb antibody (clone B-10, sc-271823 SantaCruz) whereas the lower membranes were incubated with mouse anti-β-Actin mAb (clone C4, sc-477778 SantaCruz). Proteins were visualized by ChemiDoc™ XRS+ System (BIO-RAD, California, USA) (max exposition: 600 seconds).


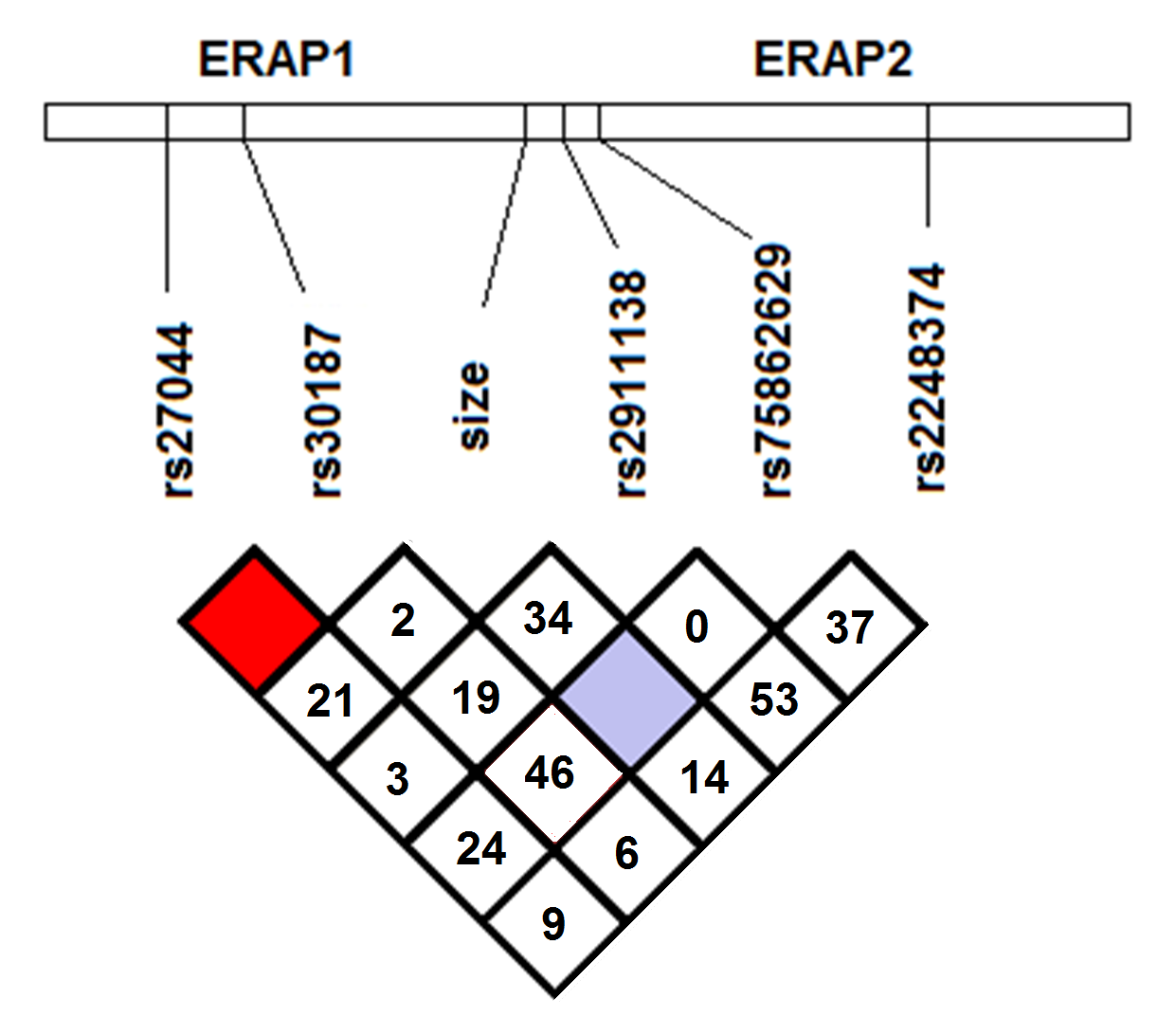


**Supplementary Figure S4.** ERAP1 and ERAP2 Linkage Disequilibrium plot of the markers analysed in the 44 B-LCLs. A standard color scheme is used to display LD with bright red color for very strong LD (LOD = 2 D' = 1), white color for no LD (LOD<2, D'<1), pink red (LOD = 2 D'<1), and blue (LOD<2 D' = 1) for intermediate LD.

**Table S1.** Oligonucleotides used for specific amplifications before sequencing analysis and for specific amplifications in *ERAP1* and *ERAP2* expression analysis. M1, M2 and M3 correspond to genomic regions (5q15) indicated in Figure S5.

| Target | Annealing °C | PCR product lenght (bp) | Forward | Reverse |
| --- | --- | --- | --- | --- |
|  |  |  |  |  |
| M1 | **58** | **739** | **5'-CTCCATGACAGTTTACAAATGCC-3'** | **5'-CTGACAAGTCCACCAAGCCT-3'** |
| CA repeats | **60** | **231** | **5'-GGCTTGGTGGACTTGTCAG-3'** | **5'[6FAM]-TGCACGCCTTTCTTGAAC-3'** |
| M2 | **57** | **767** | **5'-CTGGGAAGACATGATTGAAGG-3'** | **5'-TCCACAAAGATGAAACCTCTTG-3'** |
| M3 | **57** | **808** | **5'-ACATCTTAATGTTACCAGCGGA-3'** | **5'-AAAGAATGTTACCTCTGGCTC-3'** |
|  |  |  |  |  |
| ERAP1 | **60** | **178** | **5'-ATTCTTACACTCATTGGCAG-3'** | **5'-ATCCTTTTACCTCTTCAAGC-3'** |
| ERAP2 | **60** | **135** | **5'-GAAGTTTCCTATAACAAGGGAG-3'** | **5'-CCACAAGTCATCATTCTTAGC-3'** |
| β-Actin | **60** | **131** | **5'-GACGACATGGAGAAAATCTG-3'** | **5'-ATGATCTGGGTCATCTTCTC-3'** |

**Table S2.** B-LCLs genotyping of polymorphisms included in this study. The polymorphism reported as “size” referred to microsatellite (CA repeats) in the *ERAP1* gene promoter (~100 bp upstream 5’UTR). Allele “S”< 32 repeats; allele “L”≥ 32 repeats.

|  | rs27044 | | rs30187 | | SIZE | | rs2911138 | | rs75862629 | | rs2248374 | |
| --- | --- | --- | --- | --- | --- | --- | --- | --- | --- | --- | --- | --- |
| A | **C** | **C** | **C** | **T** | **S** | **S** | **C** | **T** | **G** | **G** | **A** | **G** |
| B | **G** | **G** | **T** | **T** | **S** | **L** | **T** | **T** | **A** | **G** | **A** | **G** |
| C | **G** | **G** | **T** | **T** | **S** | **S** | **T** | **T** | **A** | **G** | **A** | **G** |
| D | **C** | **C** | **C** | **T** | **S** | **L** | **T** | **T** | **A** | **G** | **A** | **G** |
| E | **G** | **G** | **T** | **T** | **S** | **L** | **C** | **T** | **A** | **G** | **A** | **G** |
| F | **G** | **G** | **T** | **T** | **S** | **S** | **T** | **T** | **A** | **A** | **G** | **G** |
| G | **C** | **C** | **C** | **T** | **S** | **S** | **T** | **T** | **A** | **G** | **A** | **G** |
| H | **C** | **G** | **T** | **T** | **S** | **S** | **T** | **T** | **A** | **G** | **A** | **G** |
| I | **C** | **G** | **T** | **T** | **S** | **S** | **T** | **T** | **A** | **G** | **A** | **A** |
| J | **C** | **G** | **C** | **T** | **S** | **S** | **T** | **T** | **A** | **G** | **A** | **A** |
| K | **G** | **G** | **T** | **T** | **S** | **L** | **C** | **C** | **A** | **A** | **G** | **G** |
| L | **G** | **G** | **T** | **T** | **S** | **S** | **C** | **T** | **A** | **A** | **G** | **G** |
| M | **C** | **C** | **C** | **T** | **S** | **S** | **T** | **T** | **A** | **A** | **A** | **G** |
| N | **C** | **G** | **T** | **T** | **S** | **S** | **T** | **T** | **A** | **A** | **G** | **G** |
| O | **C** | **C** | **C** | **C** | **S** | **S** | **C** | **C** | **G** | **G** | **G** | **G** |
| P | **C** | **C** | **C** | **C** | **S** | **L** | **C** | **T** | **A** | **A** | **A** | **G** |
| Q | **C** | **C** | **C** | **C** | **S** | **S** | **T** | **T** | **A** | **A** | **A** | **G** |
| R | **C** | **G** | **C** | **T** | **S** | **S** | **T** | **T** | **A** | **A** | **A** | **G** |
| S | **C** | **C** | **C** | **C** | **S** | **S** | **C** | **T** | **A** | **A** | **A** | **A** |
| T | **C** | **G** | **C** | **T** | **S** | **S** | **T** | **T** | **A** | **A** | **A** | **G** |
| U | **C** | **C** | **C** | **C** | **S** | **L** | **C** | **T** | **A** | **A** | **A** | **G** |
| V | **G** | **G** | **T** | **T** | **S** | **S** | **T** | **T** | **A** | **G** | **A** | **A** |
| W | **C** | **G** | **C** | **T** | **S** | **L** | **C** | **T** | **A** | **A** | **A** | **G** |
| X | **C** | **C** | **C** | **C** | **S** | **S** | **C** | **C** | **A** | **A** | **A** | **G** |
| Y | **C** | **C** | **C** | **T** | **S** | **S** | **C** | **T** | **A** | **A** | **A** | **G** |
| Z | **G** | **G** | **T** | **T** | **S** | **S** | **T** | **T** | **G** | **G** | **A** | **A** |
| AI | **C** | **C** | **C** | **T** | **S** | **S** | **T** | **T** | **A** | **A** | **A** | **G** |
| BI | **C** | **C** | **C** | **T** | **S** | **S** | **T** | **T** | **A** | **A** | **A** | **A** |
| CI | **C** | **G** | **C** | **T** | **S** | **S** | **T** | **T** | **A** | **A** | **A** | **G** |
| DI | **C** | **C** | **C** | **C** | **S** | **L** | **T** | **T** | **A** | **A** | **A** | **A** |
| EI | **G** | **G** | **T** | **T** | **L** | **L** | **C** | **T** | **A** | **A** | **A** | **G** |
| FI | **C** | **G** | **C** | **T** | **L** | **L** | **C** | **T** | **A** | **A** | **A** | **G** |
| GI | **C** | **G** | **C** | **T** | **S** | **L** | **C** | **T** | **A** | **A** | **G** | **G** |
| HI | **C** | **C** | **C** | **T** | **S** | **L** | **T** | **T** | **A** | **A** | **A** | **G** |
| II | **C** | **G** | **C** | **T** | **S** | **S** | **T** | **T** | **A** | **A** | **A** | **G** |
| JI | **C** | **G** | **C** | **T** | **S** | **S** | **T** | **T** | **A** | **A** | **G** | **G** |
| KI | **C** | **C** | **C** | **T** | **S** | **S** | **T** | **T** | **A** | **A** | **A** | **A** |
| LI | **C** | **G** | **C** | **T** | **S** | **L** | **T** | **T** | **A** | **A** | **A** | **A** |
| MI | **C** | **G** | **C** | **T** | **S** | **L** | **C** | **T** | **A** | **A** | **A** | **G** |
| NI | **C** | **C** | **C** | **C** | **S** | **L** | **T** | **T** | **A** | **A** | **A** | **G** |
| OI | **C** | **G** | **T** | **T** | **S** | **S** | **C** | **T** | **A** | **A** | **G** | **G** |
| PI | **C** | **C** | **C** | **C** | **S** | **S** | **T** | **T** | **A** | **A** | **G** | **G** |
| QI | **C** | **C** | **C** | **C** | **S** | **S** | **T** | **T** | **A** | **A** | **G** | **G** |
| RI | **C** | **G** | **C** | **T** | **S** | **L** | **C** | **T** | **A** | **A** | **A** | **A** |
